# Thrombotic mechanisms in low-thrombogenicity mechanical mitral valve prostheses

**DOI:** 10.1093/ehjci/jeac221

**Published:** 2022-11-10

**Authors:** Jeorghino Lodge, Alex Pitcher, Vivek Srivastava, Marco Spartera

**Affiliations:** Cardiology, Great Western Hospital NHS Foundation Trust, Marlborough Road, Swindon, SN3 6BB, UK; Cardiothoracic Department, Oxford University Hospitals NHS Foundation Trust, John Radcliffe Hospital, Headley Way, Oxford, OX3 9DU, UK; Cardiothoracic Department, Oxford University Hospitals NHS Foundation Trust, John Radcliffe Hospital, Headley Way, Oxford, OX3 9DU, UK; Cardiology, Great Western Hospital NHS Foundation Trust, Marlborough Road, Swindon, SN3 6BB, UK; Cardiothoracic Department, Oxford University Hospitals NHS Foundation Trust, John Radcliffe Hospital, Headley Way, Oxford, OX3 9DU, UK; Division of Cardiovascular Medicine, Radcliffe Department of Medicine, University of Oxford, OCMR, Level 0, West Wing, John Radcliffe Hospital, Headley Way, Oxford OX3 9DU, UK

A 63-year-old man known for mitral valve replacement (MVR) with a bi-leaflet mechanical valve (Open Pivot Medtronic 31) one year previously, presented with chest discomfort, a new murmur, and modestly increased inflammatory markers. He had been treated with warfarin with satisfactory home- and laboratory-monitored INR levels since the operation. In particular, he had several INR measurements in the preceding 3 months which had all been within the therapeutic range.

**Figure:**
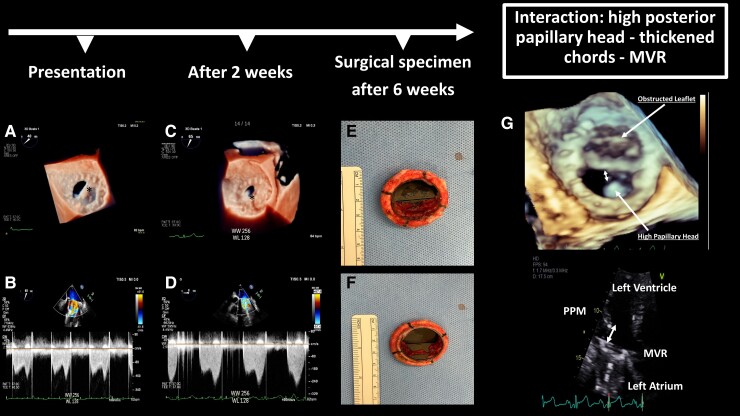


A transoesophageal echocardiogram (TOE) revealed a non-mobile mass adjacent to the posteromedial occluder causing restricted opening and partial obstruction of the MVR (*Panels A* and *B*; *Videos 1* and *2*). Blood cultures were negative including for fastidious microorganisms. Treatment with unfractionated heparin and antibiotics was given empirically for 2 weeks, and although inflammatory markers normalized, repeat TOE showed a progression to severe MVR obstruction with increased mass size now partially restricting also the anterolateral occluder (*Panels C* and *D*; *Videos 3* and *4*). Persistent obstruction on transthoracic imaging was subsequently demonstrated and re-sternotomy surgery was therefore advised. Surgical inspection revealed a laminated thrombus on the posteromedial occluder of the MV prosthesis (*Panels E* and *F*).

We report partial thrombosis and obstruction of a mechanical MVR with inherently low thrombogenicity profile despite demonstrably adequate anticoagulation. We hypothesize that the unusual anatomical interaction between a high posterior papillary muscle head, thickened chords, and the MVR led to locally turbulent flow and thrombus formation in the posteromedial region of the prosthesis (*Panel G*). Unveiling pro-thrombotic mechanisms is paramount for secondary prevention: surgical reconstruction of the papillary muscle head was performed alongside redo MVR surgery.

*Panel A*: Three-dimensional zoom showing mass (*) on the atrial side of the mechanical mitral valve with the partial opening of the posteromedial leaflet due to an obstructive mass. *Panel B*: Continuous wave Doppler across the MV showing increased gradients. *Panel C*: Three-dimensional zoom showing completely obstructed posteromedial occluder and partially restricted anterolateral occluder due to enlarging mass (*) on the atrial side of the MVR. *Panel D*: Continuous wave Doppler showing worsening mitral stenosis across the partially occluded mechanical mitral valve. *Panels E* and *F*: Surgical inspection of the excised MV prosthesis revealed a laminated thrombus on the atrial surface (*E, corresponding to the TOE imaging*) and ventricular one (*F*). *Panel G*: Three-dimensional zoom and 2D transthoracic echocardiography (three-chamber) image showing complex interaction between high posterior papillary muscle head (PPM), thickened chords, and a very close MVR (distance = double arrow) which likely led to turbulent flow locally and thrombus formation in the posteromedial region of the prosthesis.

## Data Availability

The data underlying this article will be shared on reasonable request to the corresponding author.

